# Oil body bound oleosin-rhFGF9 fusion protein expressed in safflower (*Carthamus tinctorius* L.) stimulates hair growth and wound healing in mice

**DOI:** 10.1186/s12896-018-0433-2

**Published:** 2018-08-29

**Authors:** Jingbo Cai, Ruicheng Wen, Wenqing Li, Xiuran Wang, Haishan Tian, Shanyong Yi, Linbo Zhang, Xiaokun Li, Chao Jiang, Haiyan Li

**Affiliations:** 1 College of Life Sciences, Engineering Research Center of the Chinese Ministry of Education for Bioreactor and Pharmaceutical Development, Jilin Agricultural University, Changchun, Jilin, 130118 China; 2 College of Life and Environmental Sciences, Wenzhou University, Wenzhou, Zhejiang, 325035 China; 3 Wenzhou Biomedical Innovation Center, Wenzhou University, Wenzhou, Zhejiang, 325035 China

**Keywords:** Human fibroblast growth factor 9, Safflower (*Carthamus tinctorius* L.), Oil body, Hair growth, Wound healing, β-catenin

## Abstract

**Background:**

Fibroblast growth factor 9 (FGF9) is a heparin-binding growth factor, secreted by both mesothelial and epithelial cells, which participates in hair follicle regeneration, wound healing, and bone development. A suitable source of recombinant human FGF9 (rhFGF9) is needed for research into potential clinical applications. We present that expression of oleosin-rhFGF9 fusion protein in safflower (*Carthamus tinctorius* L.) seeds stimulates hair growth and wound healing.

**Results:**

The oleosin-rhFGF9 expressed in safflower seeds, in which it localizes to the surface of oil bodies. The expression of oleosin-rhFGF9 was confirmed by polyacrylamide gel electrophoresis and western blotting. According to BCA and Enzyme-linked immunosorbent assay (ELISA) assay, the results show that the expression level of oleosin-rhFGF9 was 0.14% of oil body protein. The oil body bound oleosin-rhFGF9 showed mitogenic activity towards NIH3T3 cells in a methylthiazolyldiphenyl-tetrazolium bromide (MTT) assay. The efficacy of oil body bound oleosin-rhFGF9 in promoting hair growth and wound healing was investigated in C57BL/6 mice. In a hair regeneration experiment, 50 μg/μl oil body bound oleosin-rhFGF9 was applied to the dorsal skin of mice in the resting phase of the hair growth cycle. After 15 days, thicker hair and increased number of new hairs were seen compared with controls. Furthermore, the number of new hairs was greater compared with rhFGF9-treated mice. The hair follicles of mice treated with oil body bound oleosin-rhFGF9 expressed β-catenin more abundantly. In a wound healing experiment, dorsal skin wounds were topically treated with 50 μg/μl oil body bound oleosin-rhFGF9. Wound healing was quicker compared with mice treated with rhFGF9 and controls, especially in the earlier stages of healing.

**Conclusions:**

The oil body bound oleosin-rhFGF9 promotes both hair growth and wound healing. It appears to promote hair growth, at least in part, by up-regulating β-catenin expression. The potential of oil body bound oleosin-rhFGF9 as an external drug can treat the alopecia and wounds or use in further clinical application.

**Electronic supplementary material:**

The online version of this article (10.1186/s12896-018-0433-2) contains supplementary material, which is available to authorized users.

## Background

Fibroblast growth factor 9 (FGF9), also known as Glia-activating factor (GAF), was originally isolated from human glioma cells [1, 2]. FGF9, FGF16 and FGF20 are similar in structure and comprise the FGF9 subfamily of the fibroblast growth factor (FGF) superfamily. Full-length human FGF-9 is a 208 amino acid polypeptide lacking a typical signal sequence. Accordingly, the secretion of FGF9 is mediated by an N-linked carbohydrate chain and occurs via the constitutive endoplasmic reticulum/Golgi secretory pathway [2, 3]. The human *FGF9* gene is located on chromosome 13q11-q12, whereas mouse *FGF9* is located on chromosome 14; the human and mouse coding sequences exhibit 88.7% identity [4, 5]. There are four tyrosine kinase fibroblast growth factor receptors (FGFR1–4), which undergo alternative splicing to produce isoforms with high affinity, ligand-dependent responses to different FGFs. The specificity of FGF ligand receptor interaction is augmented by heparin, heparin sulfate, or other glycosaminoglycan chains to ensure stable binding and to activate diverse physiological functions. FGF9 can bind and activate FGFR3IIIc, FGFR3IIIb and FGFR2IIIc, but can also activate other receptors with lower affinity, such as FGFR1IIIc and FGFR4; FGF9 has highest affinity to bind and activate FGFR3 but shows no activity toward FGFR1b or -2b [6, 7].

FGF9 is found in a wide variety of tissues and organs and has multiple physiological functions. It is an indispensable growth factor for human development. FGF9 contributes to bone development and repair, angiogenesis, embryonic development, cell apoptosis, nerve regeneration, and hair follicle regeneration [8–10]. In addition, FGF9 participates in the development of heart, brain, kidney, muscle, joint and other tissues [11].

The deletion or overexpression of FGF9 causes many related diseases, such as major depressive disorder [12], multiple synostoses syndrome [13], elbow knee synostosis [14], disorders of sex development, primary synovial chondromatosis, Dupuytren’s disease [15], skeletal dysplasia, and colorectal, endometrial and ovarian carcinoma [16]. The widespread biological functions of FGF9 have drawn significant attention to its potential for clinical application, which has been addressed in many studies.

In particular, previous reports have focused on cancers or tumors, the treatment of bone related disorders, wound healing, and the exploration of hair regeneration. In light of ongoing research into applications of FGF9, there is a demand to produce this protein at low expense, and high efficiency and safety.

Plant expression systems and the oil body oleosin technology provide a convenient method for the production of exogenous recombinant fusion proteins in a large-scale, reliable, cheap, safe, effective and short-term manner. Oil bodies are simple storage organelles found in oil seeds, with a diameter of 0.5–2.0 μm, comprising a triacylglycerol matrix enclosed by a monolayer of phospholipids and structural oil body membrane proteins, principally oleosins [17]. Markley et al. [18] used this approach to express recombinant proteins in the oil bodies of transgenic seeds. Recombinant proteins were targeted to the surface of oil bodies through covalent fusion with oleosin.

The oil bodies of safflower (*Carthamus tinctorius* L.) were chosen for the production of rhFGF9. Safflower is a small acreage crop that is largely self-pollinating with low out-crossing habits and genetic stability, and is well adapted to the semi-arid conditions of the tropics and subtropics [18, 19]. An annual plant from the family Compositae, safflower can be grown counter-seasonally with cost-effective large-scale production, allowing maximum flexibility for the management of seed transport and storage at normal atmospheric temperature. As an important oil seed crop, safflower contains high oil content in seeds, ranging from 28 to 30%, which is increased 5–8% in improved varieties [20].2The seeds contain a large number of oil bodies but small amounts of water, which ensures oil body stability. The oil bodies can easily be extracted from seeds by grinding, separating by centrifugation and then purifying by washing, to provide an economical, convenient and fast procedure for obtaining exogenous proteins.

Hair loss (alopecia) is a common phenomenon and can be emotionally troubling to affected people. Minoxidil and finasteride are currently used to treat alopecia. Both slow the progression of hair loss but do not regenerate new hair, and both can cause side effects. Growth factors are potential therapeutic agents that could reduce side effects and create the possibility of hair regrowth, leading to studies of their functions and mechanisms of action. Gay and colleagues [9] studied the molecular mechanisms of hair follicle regeneration in a wound-induced hair neogenesis model, suggesting that FGF9 from γδ T cells modulates hair follicle regeneration and triggers Wnt expression and subsequent Wnt activation in wound fibroblasts through a unique feedback mechanism. Treatment with a growth factor cocktail including FGF9, delivered by microneedling, was effective and safe, and seemed to be more effective than a growth factor cocktail lacking FGF9 [21]. It has been reported that FGFs stimulate hair growth through β-catenin and Sonic hedgehog (Shh) expression [22]. Martz et al. [23] revealed that the FGF9-mediated promotion of hair regeneration is related to the Wnt pathway. In addition, FGF9 is secreted by both mesothelial and epithelial cells, and plays important roles in organ development [24, 25]. In young mice, FGF9 mRNAs were increased on day 2 after wounding compared with day 0, and were significantly upregulated at other times during wound healing [26]. Evaluation of FGF9 expression during the healing of mouse and adult human skin following laser ablation showed that the expression of FGF9 protein and mRNA were up-regulated [27]. Thus, FGF9 has potential therapeutic application for promoting both hair growth and wound healing.

## Results

### Construction of rhFGF9 expression vector

The rhFGF9 expression vector was constructed and showed as previously report [8]. And clones carrying the desired expression vector were identified by PCR to evaluate insert size and verified by restriction enzyme digestion.

### Safflower transformation and transgenic plant regeneration via grafting

**Fig. 1:**
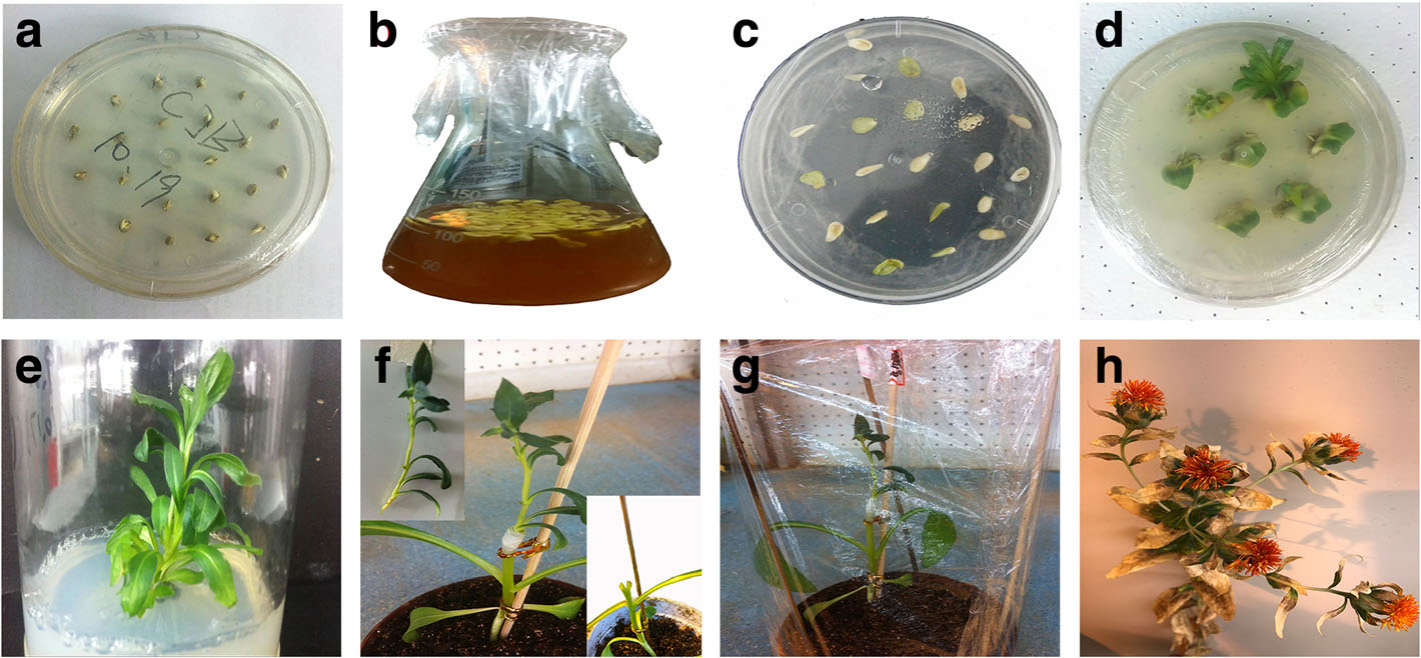
*Agrobacterium-mediated* transformation of safflower and transgenic plants of safflower via tissue culture and grafting. **a** Seeds germination. **b** Infection with Agrobacterium. **c** Co-cultivation. d Shots initiation. **e** Seedlings elongation. **f** grafted plantlets with parafilm holding V-shaped transgenic scion and rootstock by grafting. **g** The successful grafted plant in pot through covering with preservative film. **k** The mature T_0_ transgenic plant

The pOTBar-oleosin-rhFGF9 plasmid was transformed into the safflower genome using the *Agrobacterium tumefaciens*-mediated infection method, and grafted transgenic safflower plants were obtained (Fig. 1). The numbers of plants transformed and grafted at major steps in the procedure are listed in the animation (Table 1). The transformation rate was 0.75%.

**Table 1:** Plants transformation and grafting of significant steps and quantity statistics

Cotyledon explants	Shoots as scions	Survived grafts	Transgenic plants	Transformation efficiency^a^
1869	242	86	14	0.75%

^a^The transformation efficiency was calculated as the (transgenic plants/cotyledons) × 100%

**Fig. 2:**
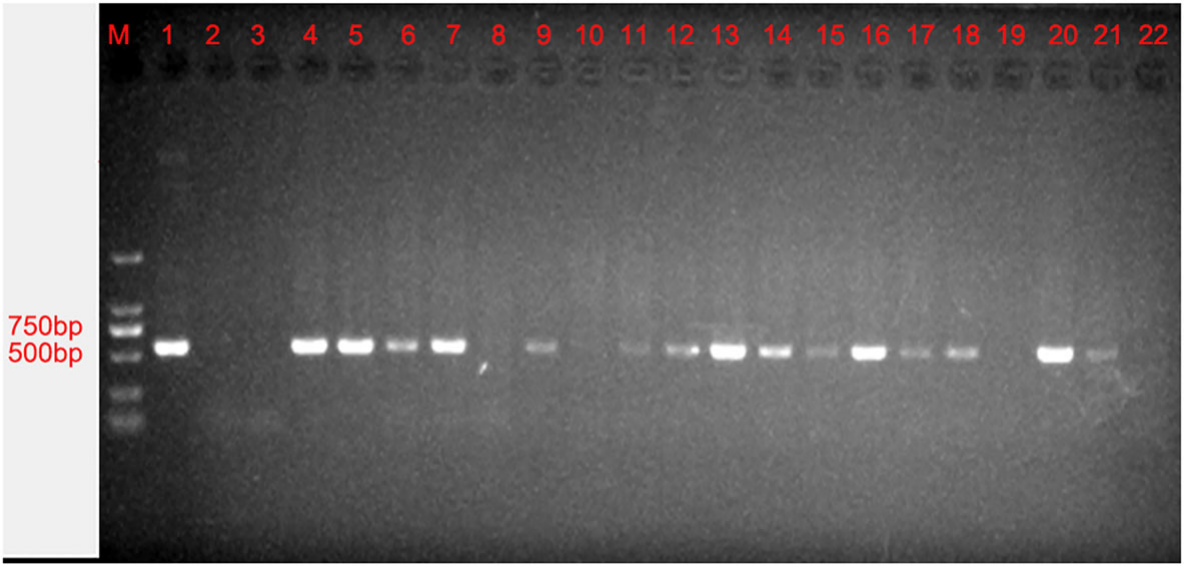
T_0_ safflower plants PCR amplification products of genomic DNA for 1% agarose gel. M: 2000 DNA maker. Lane 1: positive control (plasmid pOTBar-oleosin-rhFGF9). Lane 2: blank control (ddH_2_O). Lane 3: negative control (wild-type safflower). Lane 4–22: PCR amplification products of successful grafted safflower plants

Successfully grafted T_0_ safflower plants were identified by PCR analysis of genomic DNA to determine whether oleosin-rhFGF9 had integrated into the genome. Fourteen transgenic safflower plant lines were identified and harvested from 15 independent infections (Fig. 2). T_1_ and T_2_ plants were produced in agreement with Mendel’s law of segregation. Plants without the oleosin-rhFGF9 gene, identified by PCR, were discarded.

### Protein analysis of oleosin-rhFGF9

**Fig. 3:**
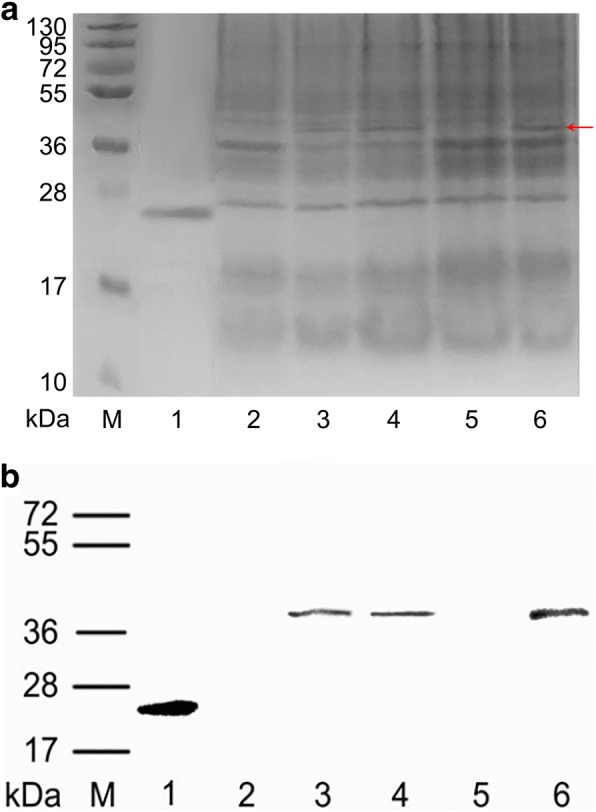
SDS-PAGE and Western blotting of protein analysis of oleosin-rhFGF9. **a** Identification of oleosin-rhFGF9 from transgenic safflower by SDS-PAGE. M: Protein Marker. Line 1: purified rhFGF9. Lane 2: oil body protein of the wild-type (WT) safflower. Lane 3–6: oil body protein of the transgenic safflower (T_3_–2, T_3_–4, T_3_–10, T_3_–17). **b** Western blotting analysis on oleosin-rhFGF9 from the transgenic safflower. M: Protein Marker. Line 1: purified rhFGF9. Lane 2: oil body protein of the wild-type (WT) safflower. Lane 3–6: oil body protein of the transgenic safflower (T_3_–2, T_3_–4, T_3_–10, T_3_–17)

The oil body-bound oleosin-rhFGF9 fusion protein expressed in transgenic T_3_ safflower seeds was evaluated by SDS-PAGE and western blotting (Fig. 3). A band of 41.5 kDa was seen for transgenic T_3_ safflowers but not for WT (wild type) safflowers. The molecular weight and position of this band corresponded to an FGF9-positive band identified by western blotting. And according to BCA and ELISA assay, the results show that the level of oil body protein was 2.4% of safflower seeds, and the expression level of oleosin-rhFGF9 was 0.14% of oil body protein.

### Mitogenic activity of oleosin-rhFGF9

**Fig. 4:**
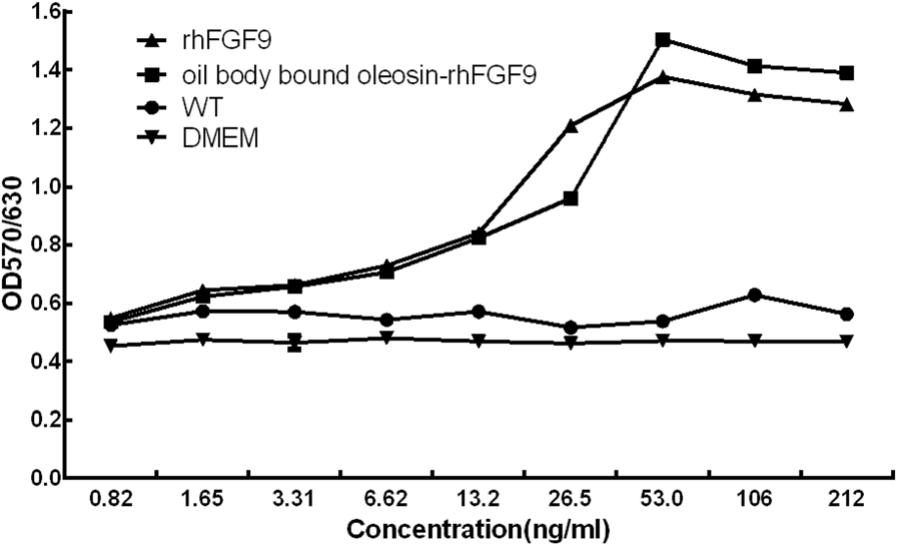
The effect of oil body bound oleosin-rhFGF9 of transgenic safflower was analyzing the activity of NIH/3 T3 cells. Various concentrations (0.82–212 ng/ml) of wild-type (WT) and oil body bound oleosin-rhFGF9 or rhFGF9 (FGF9 from E.coli) were used in NIH/3 T3 cells. DMEM was used as a blank control. Proliferation was quantified by measuring the absorbance at 570/630 nm

The effect of oil body bound oleosin-rhFGF9 from transgenic T_3_ safflowers on NIH/3 T3 cell proliferation was examined using a standard MTT method. The proliferative activity induced by oil body bound oleosin-rhFGF9 was comparable with that induced by the positive control (purified rhFG9) (Fig. 4). Furthermore, a dose-dependent effect of oil body bound oleosin-rhFGF9 on cell proliferation was seen. The oil bodies of wild-type (WT) safflower also increased proliferation, but this effect was not statistically significant. Only rhFGF9 and oil body bound oleosin-rhFGF9 significantly increased the proliferation of NIH/3 T3 cells.

### In vivo analysis of oleosin-rhFGF9 activity

**Fig. 5:**
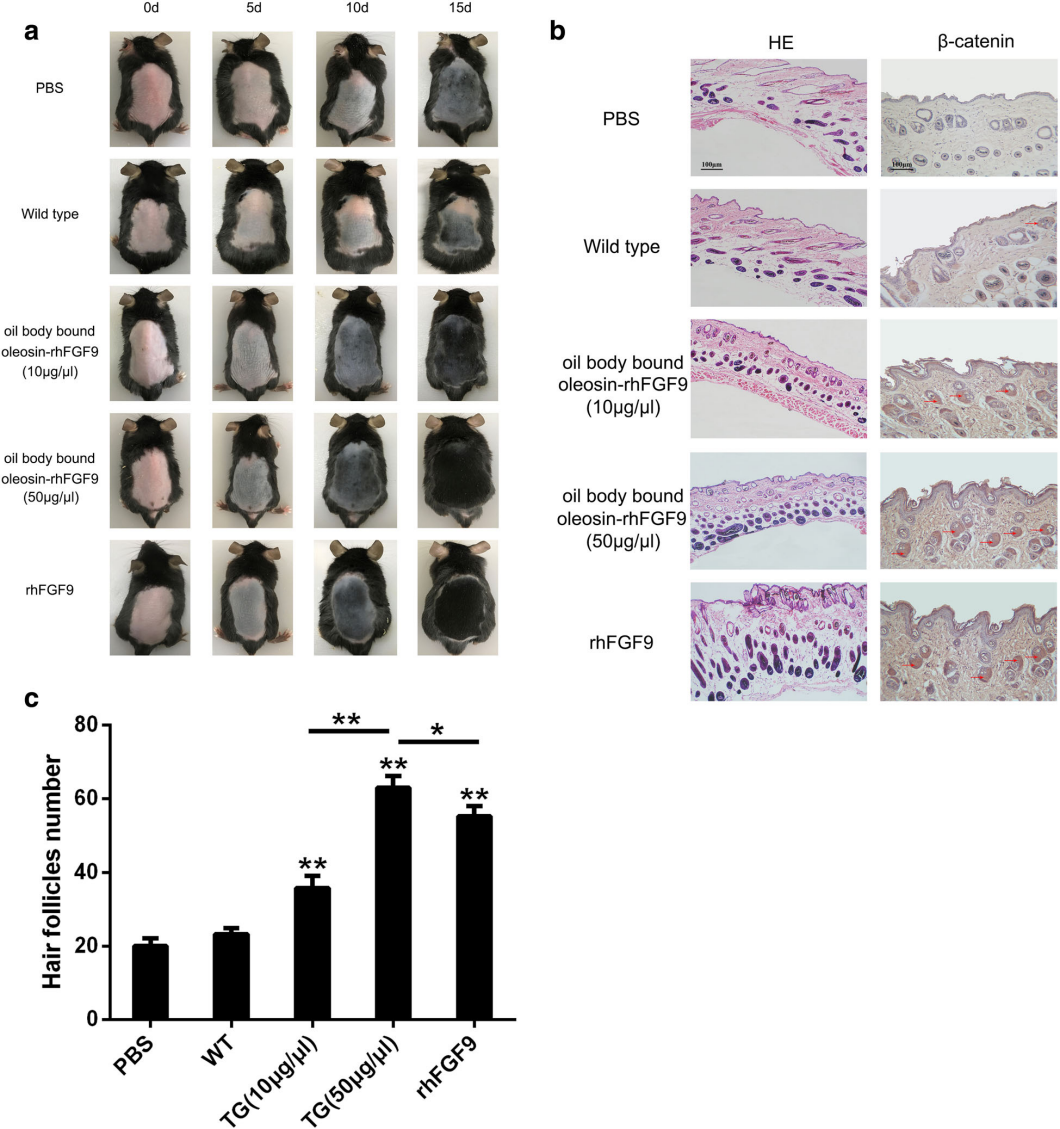
The effect of the oil body bound oleosin-rhFGF9 of transgenic safflower induces hair growth after depilation when hair follicle was telogen. **a** oil body bound oleosin-rhFGF9 was daubed on the dorsal skin of C57BL/6 mice (6-week-old). On every 0, 5, 10, 15 day, C57BL/6 mice was taken photographs and observed hair growth status. **b** Sections of dorsal skin were stained by HE and the expression of β-catenin was detected by immunohistochemical staining. Brown represents positive result with red arrow. Scale bars = 100 μm. c The number of hair follicles in skin. Data are the mean ± SD, *P < 0.05, **P < 0.01 compare to blank control group. TG represents treated group with oil body bound oleosin-rhFGF9

**Fig. 6:**
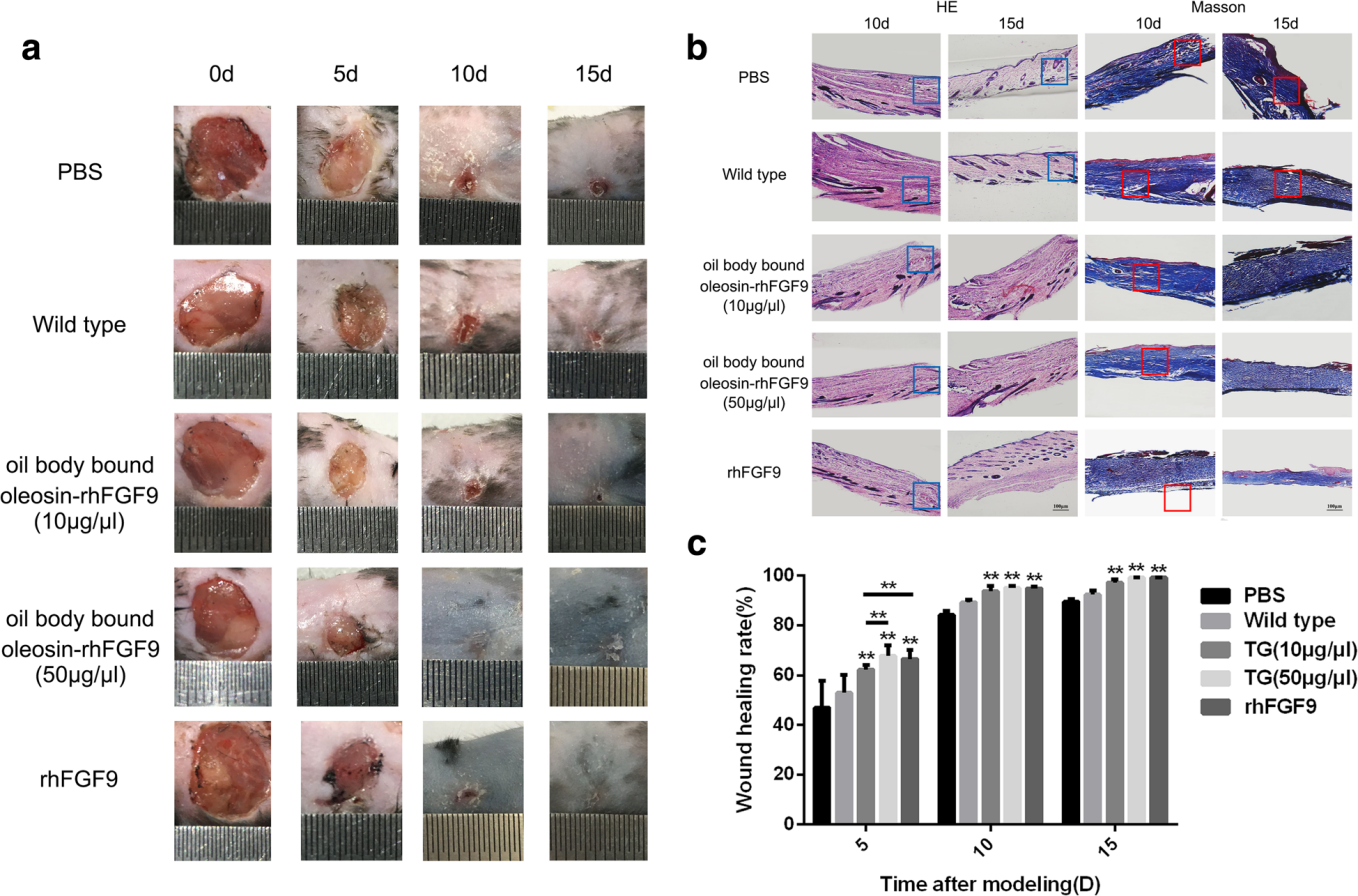
The effect of the oil body bound oleosin-rhFGF9 of transgenic safflower induces wound healing. **a** Oil body bound oleosin-rhFGF9 was daubed on the wound skin of C57BL/6 mice (6-week-old). On every 0, 5, 10, 15 day, C57BL/6 mice was taken photographs and observed wound healing rate. **b** Sections of dorsal skin were stained by HE and Masson. Scale bars = 100 μm. c The rate of wound healing in skin. Data are the mean ± SD, *P < 0.05, **P < 0.01 compare to blank control group. TG represents treated group with oil body bound oleosin-rhFGF9

C57BL/6 mice were used to investigate the effects of oil body bound oleosin-rhFGF9 on both hair growth and wound healing. At the beginning of the experiments, the hair follicles of depilated dorsal skin were in the resting phase of the hair cycle (telogen), and so the skin was glossy and pink. After 15 days of treatment, oil body bound oleosin-rhFGF9 showed obvious effects in both the hair regeneration (Fig. 5) and wound healing experiments (Fig. 6). In the hair regeneration experiment, dorsal skin had darkened on day 5, indicating that the growth phase of the hair cycle (anagen) had begun. The skin was gray with extremely short hair shafts emerging on day 10. The skin was black with many new hairs visible on day 15. Overall, the positive control group and the two oil body bound oleosin-rhFGF9 treated groups exhibited more visible hair growth over several days compared with the blank control group and the negative control group. Moreover, the high dose of oil body bound oleosin-rhFGF9 (50 μg/μl) had a greater effect on hair growth than rhFGF9 (0.07 μg/μl) or the low dose of oil body bound oleosin-rhFGF9 (10 μg/μl). Hair regrowth was slow in the blank control and negative control groups. The numbers of regenerating of hair follicles seen in H&E stained sections were consistent with the macroscopically-observed hair regrowth results (Fig. 5b, c). The expression of β-catenin in skin was localized to hair follicles (Fig. 6b). Stronger β-catenin staining was seen following treatment with oil body bound oleosin-rhFGF9 (50 μg/μl) compared with the other groups.

In the wound healing experiment, healing had obviously begun on day 5, the wounds were almost completely healed on day 10, and the wounds were fully healed with a small white scar visible on day 15. Overall, the positive control group and the two oil body bound oleosin-rhFGF9 treated groups exhibited faster wound healing compared with the blank control and negative control groups. The high dose of oil body bound oleosin-rhFGF9 (50 μg/μl) had a greater effect on wound healing than rhFGF9 (0.07 μg/μl) or the low dose of oil body bound oleosin-rhFGF9 (10 μg/μl). This difference was significant on day 5, but the difference to rhFGF9 was not significant on day 10 and 15. The blank control and negative control groups showed slow wound healing. The degree of wound healing seen in H&E and Masson’s trichrome stained sections was consistent with the results described above (Fig. 6b). The rates of wound healing are shown in Fig. 6c.

## Discussion

Clinical applications of FGFs have been extensively studied, including FGF9, and there is a need for a suitable supply of recombinant FGF proteins. At present, three major expression systems are used to produce proteins, *Escherichia coli*, insect cells and animal cells, but all have important limitations. Plant expression systems offer distinct advantages, including economy, time savings, convenience, safety, long storage, easy transportation and high yield [28]. The use of safflower as a plant expression system for the production of a oleosin-rhFGF9 fusion protein offers maximum benefit. Moreover, transgenic oil body-oleosin technology is already widely employed in the model plant, *Arabidopisis thaliana* and in safflower [18]. Without the need for tedious, difficult purification, fusion proteins in oil bodies are easily accessible for topical application to the skin.

In this study, we successfully constructed an oleosin-rhFGF9 expression vector, transformed safflower using the *Agrobacterium tumefaciens*-mediated method, and expressed oleosin-rhFGF9 in the oil bodies of transgenic seeds. According to BCA and ELISA assay, the results show that the level of oil body protein was 2.4% of safflower seeds, and the expression level of oleosin-rhFGF9 was 0.14% of oil body protein; 140.66 g oleosin-rhFGF9 can be produced in 1 ha of transgenic safflowers, and 4200 kg of safflower per hectare. The oil body bound oleosin-rhFGF9 was biologically active. We found that it had notable, dose-dependent mitogenic activity towards NIH/3 T3 cells. The greatest mitogenic activity was seen at a concentration of 53 ng/ml. Importantly, the oil body bound oleosin-rhFGF9 also had significant effects on hair growth and wound healing, and appeared to induce hair growth through the up-regulation of β-catenin.

As an appendageal organ of skin, hair plays an important role in temperature regulation and physical protection. The hair follicle, a unique, characteristic organ of mammals, represents a stem cell-rich, prototypic neuroectodermal-mesodermal interaction system. The hair follicle is composed of epidermis and dermis, which contain the root sheaths and dermal papilla respectively. The regrowth of hair requires signaling between the epidermal and dermal components [29]. An increasing number of people are affected by serious skin diseases such as alopecia, cracked skin and varying degrees of burns. FGF9 is secreted by both mesothelial and epithelial cells, and only is expressed in the epithelium. It is involved in the early differentiation of epithelial cell layers, in epithelial invagination and ectodermal organogenesis (ectodermal organs include hair, feathers, scales, teeth, beaks, nails, horns and several eccrine glands) [27, 30]. In hair follicles, FGF9 mRNA expression is highest in telogen, at 22 days after depilation, at approximately 300 mRNA copy numbers per cell; FGFR2 and FGFR3 are abundantly expressed in anagen VI (at 18 days), at approximately 10,000 and 28,000 mRNA copy numbers per cell respectively [31]. Compared with other FGFs (FGF1, 2, 5, 7, 10, 13, and 22), FGF9 is expressed in skin at a relatively low mRNA copy number. However, FGF9 from dermal γδ T cells induces hair follicle neogenesis after wounding, and activates Wnt expression in the wound through a unique feedback mechanism to promote skin regeneration [9]. FGF9 may induce hair growth from follicles by binding FGFR2 and FGFR3, activating downstream signaling pathway proteins, and stimulating the follicles to initiate hair regeneration. In addition, FGF9 plays a role in wound healing, and its expression is up-regulated in laser-induced wounds [27]. Thus, FGF9 has a positive effect on both hair growth and wound healing through one or two signaling pathways.

Other FGFs have previously been expressed in plant oil bodies. For example, recombinant KGF2 has been expressed in *Arabidopsis thaliana* oil bodies, and has effects on hair growth in mice [32]. FGFs stimulate hair growth in C57BL/6 mice at a concentration of 500 μg/ml [33]. When we treated mouse dorsal skin with oil body bound oleosin-rhFGF9 at 50 μg/μl and 10 μg/μl, the high dose had a notable effect on hair growth, slightly greater than that of rhFGF9 (0.07 μg/μl). Oil bodies help to maintain protein stability. The surfactant properties of oleosins and the non-coalescing nature of oil bodies mean they act as emulsifying agents, enhancing their potential for biotechnological applications [28, 34]. The absorption of topically-applied oil body bound oleosin-rhFGF9 may have been accelerated because of this emulsifying property. Abundant oil body bound oleosin-rhFGF9 can be absorbed to concentrate in the epidermis, and then penetrate into the dermis where it can act on follicles to induce hair growth.

The formation of hair requires intercellular signaling to trigger gene expression changes in the follicle. The Wnt signaling pathways play important roles in hair follicle development. Specific Wnts maintain anagen-phase gene expression in vitro and hair-inductive activity in a skin reconstitution assay [35]. The Wnt signaling pathways that are active in hair follicle development and growth cycle. FGFs promoted hair growth by inducing the anagen phase in telogen C57BL/6 mice, and FGF treatment induces the expression of β-catenin and Shh in hair follicles [33]. In this study, we found that β-catenin was expressed in hair follicles and was associated with newly growing hairs. Treatment with rhFGF9 or oil body bound oleosin-rhFGF9 (at 50 μg/μl or 10 μg/μl) resulted in stronger immunohistochemical staining compared with the negative controls, suggesting that oil body bound oleosin-rhFGF9 may promote hair growth by increasing the expression of β-catenin.

FGF9 expression is significantly upregulated at various times during wound healing in young mice, but is low in healthy skin. In particular, FGF9 mRNA is increased on day 2 after wounding, and the wound closure of aged (35-week-old) hairless mice is substantially slower than young adult (8-week-old) mice [26]. When we treated mouse dorsal skin wounds with oil body bound oleosin-rhFGF9 at 50 μg/μl and 10 μg/μl, the results were similar to the hair growth experiment. Macroscopic and histological observations indicated that the high dose of oil body bound oleosin-rhFGF9 had a notable effect on wound healing, slightly greater than that of rhFGF9 (0.07 μg/μl).

Hence, the oil body-bound oleosin-FGF9 fusion protein appears to accelerate wound healing as well as promoting hair growth.

## Conclusions

In this study, we constructed an optimized rhFGF9 expression vector (pOTB-oleosin-rhFGF9) and subsequently expressed oleosin-rhFGF9 in safflower oil bodies. Importantly, the oil body-bound oleosin-rhFGF9 produced from transgenic safflower seeds were found to have a significant mitogenic effect on NIH3T3 cells, and also to promote hair growth and wound healing. Furthermore, we undertook immunohistochemistry of β-catenin to investigate whether its expression was up-regulated after treatment with oil body bound oleosin-rhFGF9 to regrow new hair. This is the first report regarding the expression of active, oil body-bound oleosin-rhFGF9 for topical application to promote hair growth and wound healing, providing a data basis for the development of therapeutic applications.

## Methods

### Reagents and bacterial strains

TaKaRa LA Taq polymerase, the restriction enzymes, NcoI and HindIII, pfu DNA polymerase, and T4 DNA ligase were all purchased from Takara (Dalian, China). A polymerase chain reaction (PCR) purification kit, gel extraction kit, plasmid miniprep kit, plant genomic DNA extraction kit and bicinchoninic acid (BCA) kit were all purchased from Bio TeKe Corporation (Beijing, China). Kanamycin and rifampicin were purchased from Sigma (Hong Kong, China). Dulbecco’s modified Eagle medium (DMEM) was purchased from Hyclone (Logan, UT, USA). Methylthiazol tetrazolium (MTT) was obtained from Gentihold (Beijing, China). All primers and gene coding sequences were synthesized by Genewiz (Jiang Su, China). The expression vector, pOTBar, and the plasmids, pUC19-oleosin, *Escherichia coli* DH5α and *Agrobacterium tumefaciens* EHA105 were obtained from the Ministry of Education Engineering Research Center of Bioreactor and Pharmaceutical Development, Jilin Agricultural University.

### Construction of rhFGF9 expression vector

The pOTBar plasmid was used for the construction of rhFGF9 expression vector and the same method as previous report [8]. The Oleosin and rhFGF9 primers as following: oleosin forward, 5′- CCATGGCGGATACAGCTAGAGGAACC-3′; oleosin reverse, 5′- CTCTCCCAAAGGAGCCATAGTAGTGTGCTGGC-3′; rhFGF9 forward, 5′- GCCAGCACACTACTATGGCTCCTTTGGGAGAG-3′; rhFGF9 reverse, 5′- CCCAAGCTTAAGATTGAGAAAGGATATCCTTGT-3′.

### Safflower transformation and transgenic plant regeneration via grafting

*Agrobacterium tumefaciens*-mediated transformation of safflower and grafting of regenerated seedlings has been reported previously [19, 36]. The safflower seeds, JI HONG YI HAO, used in this study had been certified, and were supplied by the Ministry of Education Engineering Research Center of Bioreactor and Pharmaceutical Development, Jilin Agricultural University. Prior to germination, seeds were surface sterilized by soaking in 0.1% HgCl_2_ solution, and then handling under sterile conditions, by shaking for 10 min, and rinsing five times for 1 min each with sterile distilled water soon afterwards. The surface-sterilized seeds were germinated aseptically on seed germination medium as shown in the animation (see Additional file 1, Table R1, S1) [36]and incubated at 25 °C, in 24 h darkness, for 3–4 days (Fig. 1a). Wild safflower seeds to use as rootstocks were cultivated in a pot that included soil and vermiculite (three times as much soil as vermiculite by volume) for 20 days before grafting.

The day before transformation, 100 μl of *Agrobacterium* harboring the recombinant plasmid pOTBar-oleosin-rhFGF9 was taken from − 80 °C storage and added directly to 100 ml liquid YEP medium containing 50 mg/ml kanamycin and 25 mg/ml rifampicin, and was then grown overnight at 28 °C with agitation at 180 rpm. The *Agrobacterium* bacterial culture for infecting safflower was adjusted to OD600 = 0.6–0.8. Next, cotyledonary explants were isolated and inoculated by exposing them for 15 min to 100 ml *Agrobacterium* culture (Fig. 1b) with gentle agitation during the infection. The bacterial fluid was then discarded, and infected explants were blotted dry on sterile filter paper and transferred to co-cultivation medium (see Additional file 1, Table R1, S2; Fig. 1c) enriched with 100 μM acetosyringone. All plates were placed in a cultivation incubator at 25 °C, in darkness, for 3 days.

Three days after co-cultivation, explants were transferred to bud initiation medium (see Additional file 1, Table R1, S3) and grown at 25 °C under the cycle of 16 h day alternating with 8 h night. After approximately 15 days, when regeneration shoots become sufficiently strong and could touch the plate cover (Fig. 1d), excised explants of the regeneration shoots were placed on seedling elongation medium (see Additional file 1, Table R1, S4) and cultured at 25 °C under a cycle of 16 h day alternating with 8 h night. Before grafting, they were screened on the same medium with 0.1% glufosinate. After several days, regenerated plantlets that emerged and were growing well were tentatively judged to be transgenic candidates, and were grafted and grown until approximately 3–4 cm long (Fig. 1e).

Prepared rootstocks were grown in pots for 14 days until they had 4–6 true leaves. To prepare scions, 3–4 cm long regenerated seedlings with a strong stem were cut with a thin knife blade on both sides of the stem, at 45° angles to form a V-shape, and were then placed in distilled water to retain moisture. Suitable rootstocks were horizontally transected above the two true leaves, and the stem was then vertically slit down the center to a depth matching the V-shaped scions. A prepared scion was inserted into the rootstock and the junction between them was wrapped using parafilm, with the necessary degree of tightness to hold them together (Fig. 1f). Grafted seedlings were grown in pots made airtight by covering with a preservative film to maintain humidity, in an environment of 21 °C, 8.5 klux, 16 h day and 8 h night. After 3–4 days’ growth, plantlets were hardened by making a hole in the preservative film to enable exposure to the external environment while simultaneously retaining the moisture required for survival, and were then grown for a further 7 days (Fig. 1g). Once the scion of grafted seedlings had grown several new leaves, the preservative film covering the pots was removed. Unsuccessful grafts were discarded, and successfully grafted T_0_ transgenic plants were ultimately harvested (Fig. 1h).

### PCR validation of transgenic safflowers

Transgenesis of successfully grafted safflower plants was verified by PCR amplification of the rhFGF9 gene. Total genomic DNA that was extracted from young leaf tissue using a rapid plant genomic DNA extraction kit was used as template with the specific primers (forward: 5’-CTTTGGGAGAGGTGGGAAACTACTT-3′; reverse: 5′- CACCTGGGACTATTCCACGGACTCG-3). The positive, negative and blank controls were the pOTBar-oleosin-rhFGF9 plasmid, WT safflower leaf total DNA and double-distilled H_2_O, respectively. The thermal profile of the PCR was: initial denaturation at 94 °C for 7 min; 30 cycles of amplification at 94 °C for 30 s, 55 °C for 45 s, and 72 °C for 90 s; and finally, extension at 72 °C for 7 min. After PCR amplification, the products were evaluated by electrophoresis in 1% agarose gels.

### Oil body extraction, purification and protein analysis

For both WT and transgenic safflower plants, the shells were stripped from two or three seeds, and they were thoroughly ground using a pestle in a 1.5 ml centrifuge tube with 200 μl phosphate buffer saline (PBS, pH 7.5). The tubes were then centrifuged at 12000×g for 5 min, and the supernatant and floating oil body phase were transferred to a new 1.5 ml centrifuge tube. The above procedure was repeated twice. The oil bodies were then washed three times with 200 μl PBS, centrifuging as above to finally collected the purified oil bodies. A BCA protein assay kit was used to measure oil body protein concentration, and then the oil bodies were stored at 4 °C for further use.

Electrophoresis loading buffer and PBS were added to the oil bodies to a protein concentration of 3.5 μg/μl, and then boiled for 10 min to denature the protein. The oil body-bound oleosin-rhFGF9 was analyzed by 12% sodium dodecyl sulfate–polyacrylamide gel electrophoresis (SDS-PAGE) using 10 μl samples per lane. Gels were visualized by Coomassie brilliant blue staining and subjected to further analysis by western blotting. Proteins were transferred to polyvinylidene fluoride membranes, blocked with blocking liquid, incubated with a rabbit anti-hFGF9 polyclonal primary antibody (1:500 dilution, Bioss, Beijing, China) and a horseradish peroxidase (HRP)-conjugated secondary antibody (1:2000, Abcam, Cambridge, MA, USA), and ultimately showed by enhanced chemiluminescence. A Human FGF9 ELISA Kit (Bioss) was used to measure oleosin-rhFGF9 concentration in the oil body protein, according to the manufacturer’s protocol.

### Mitogenic activity of oleosin-rhFGF9

The NIH/3 T3 cell line (American Type Culture Collection, Rockville, MD) was used to detect the mitogenic activity of oil body bound oleosin-rhFGF9. Briefly, cells were grown in a culture flask containing DMEM, 10% fetal bovine serum (FBS), and ampicillin and streptomycin (both 100 U/ml). Cells at the appropriate growth phase were seeded into a 96-well plate (5 × 10^3^ cells per well), cultivated under normal or cell starvation conditions for 24 h, and then treated for 48 h with oil body bound oleosin-rhFGF9 or rhFGF9 (purified rhFGF9 from E.coli) at various concentrations from 0.82–212 ng/ml. The cells were then incubated with 20 μl MTT for 4 h, the culture medium was added to 100 μl dimethyl sulfoxide and mixed to homogeneity by shaking, and optical absorbance values at 570/630 nm were measured using a microplate reader.

### In vivo analysis of oleosin-rhFGF9 activity

All animal experimentation investigation conforms with the Guide for the Care and Use of Laboratory Animals published by the US National Institutes of Health (NIH Publication No. 85–23, revised 1996). The experiment was authorized by Jilin Agricultural University ethical committee. Mice were purchased from Changchun Billion Biotechnology Limited Liability Company (Changchun, China) and allowed to adapt to their new environment with free access to water and food for 1 week. Healthy 6-week-old male C57BL/6 mice (18–22 g) were randomly assigned to either the hair regeneration or the wound healing experiment. For each experiment, six of heathy male C57BL/6 mice were randomly assigned to each of five treatment groups: a blank control group (treated with PBS), a negative control group (50 μg/μl WT safflower oil body protein), a positive control group (0.07 μg/μl of purified rhFGF9 from E.coli, provided by the School of Pharmaceutical Science, Key Laboratory of Biotechnology and Pharmaceutical Engineering, Wenzhou Medical College), a high-dose group (50 μg/μl of oil body bound oleosin-rhFGF9), and a low-dose group (10 μg/μl of oil body bound oleosin-rhFGF9). All mice were anesthetized, their dorsal hair was clipped with a shaver, and then residual hair was completely removed using a depilatory paste. For the wound healing experiment, surgical scissors were used to make two 1-cm-diameter full thickness wound, one on each side of the dorsal skin. Oil bodies were extracted every second day, and the protein concentration in each preparation was measured using the BCA protein assay kit, as above. Treatments were applied by every second day daubing 100 μl onto the dorsal skin, working from the centerline outwards. The dorsal skin was subsequently photographed. On day 15, mice were sacrificed by cervical dislocation, and the dorsal skin was collected for histological analysis. Skin samples were fixed in 4% paraformaldehyde, embedded in paraffin, serially sectioned, stained with hematoxylin and eosin (H&E, Solarbio, Beijing, China) or Masson’s trichrome (Solarbio), and observed by microscopy.

### Immunohistochemistry

Skin sections were incubated in 3% peroxidase for 10 min, rinsed twice in PBS for 3 min, heated in citrate buffer to induce epitope retrieval, blocked in bovine albumin 20 for min at room temperature, and then incubated with a rabbit anti-β-catenin antibody (1:200, Bioss) at 4 °C over night. The following day, sections were incubated with a HRP-conjugated secondary antibody (1:1000, Abcam, Cambridge, MA, USA) for 30 min, and then stained with a diaminobenzidine chromogen kit (Solarbio) until they showed a palpable brown color.

### Statistical analysis

Results are presented as mean ± standard deviation (SD). Data were analyzed by GraphPad Prism, version 6.01 software (GraphPad Software Inc., La Jolla, CA, USA), and ImageJ software (Rawak Software, Inc., Munich Stuttgart, Germany). One-way ANOVA was used to compare multiple groups at the *P* < 0.05 level of significance.

## Additional file

Additional file 1: **Table R1.** Culture medium composition of safflower. (PDF 22 kb)

## Abbreviations

ELISA: Enzyme-linked immunosorbent assay
HRP: Horseradish peroxidase
MTT: Methylthiazolyldiphenyl-tetrazolium bromide
PBS: Sodium phosphate buffer
pOTBar: p1301-promoter-oleosin-Terminator-35S-Bar-Nos
rhFGF9: Recombinant human fibroblast growth factor 9
WT: Wild safflower seeds
