# Supplementary material for: Oil body bound oleosin-rhFGF9 fusion protein expressed in safflower (Carthamus tinctorius L.) stimulates hair growth and wound healing in mice
Source: BMC Biotechnol. 2018 Aug 29;18:51. doi: 10.1186/s12896-018-0433-2 (PMC6114888; doi:10.1186/s12896-018-0433-2)
Supplement: Supplementary file 1 — Table R1. Culture medium composition of safflower. (PDF 22 kb) [file 12896_2018_433_MOESM1_ESM.pdf]

Table R1. Culture medium composition of safflower

Component (mg/l) \ Media	S1	S2	S3	S4
	Seed germination	Co-cultivation	Bud initiation	Seedling elongation
Murashige & Skoog Basal Medium with Vitamins	4330	4330	4330	4330
Thiamine.HCL	0.4	0.4	0.4	0.4
Pyridoxine.HCL	0.2	0.4	0.4	0.4
Nicotinic acid	0.5	0.4	0.4	0.4
Glycine	2	2	2	2
Inositol	100	100	100	100
Sucrose	20000	30000	30000	10000
1-Naphthaleneacetic acid (NAA)	0	1.5	1.5	0
6-Benzylaminopurine (6-BA)	0	0.5	0.5	0
AS	0	19.62	0	0
KNO3	0	0	0	3800
Agar	8000	8000	8000	8000
Ceftriaxone Sodium	0	0	100	200
Carbenicillin	0	0	200	100

*1. All media should be adjusted solution pH from 5.8 to 6.0 before adding the agar.
2. All the agents were obtained from PhytoTechnology Laboratories, USA.
